# Clinical advances in racemic epinephrine for pediatric croup: a mini-review of evidence and practice

**DOI:** 10.3389/fped.2025.1616521

**Published:** 2025-06-23

**Authors:** Yu-tong Hou, Qi Shi, Lin Zhang, Qian Cheng

**Affiliations:** ^1^ Department of Pediatrics, Hongqi Hospital Affiliated to Mudanjiang Medical University, Mudanjiang, China; ^2^ Department of Pediatric Surgery, Hongqi Hospital Affiliated to Mudanjiang Medical University, Mudanjiang, China

**Keywords:** laryngotracheobronchitis, croup, racemic epinephrine, pediatric, review

## Abstract

This mini-review critically evaluates the contemporary evidence regarding racemic epinephrine's role in pediatric croup management. As the pharmacological mainstay for acute airway obstruction, racemic epinephrine demonstrates rapid efficacy through dual α₁-adrenergic vasoconstriction and β_2_-mediated bronchodilation, achieving clinically significant Westley Croup Score reductions (2–3 points) within 30 min of administration. Current evidence establishes therapeutic equipoise between racemic and L-epinephrine formulations, though important disparities exist in global accessibility and cost-effectiveness. The transient therapeutic window (90–120 min) necessitates careful monitoring and underscores the importance of concomitant corticosteroid administration for sustained symptom control. While the safety profile remains favorable, with transient cardiovascular effects representing the primary concern, several evidence gaps persist regarding optimal retreatment intervals, viral subtype-specific responses, and long-term neurodevelopmental outcomes. Emerging research directions highlight the potential of advanced delivery systems and biomarker-guided approaches to optimize therapy. These findings collectively reinforce racemic epinephrine's position as an essential bridging intervention during the critical latency period preceding corticosteroid efficacy, while emphasizing the need for standardized protocols to ensure optimal clinical implementation across diverse healthcare settings.

## 1 Introduction

Laryngotracheobronchitis (croup) represents a clinically significant pediatric respiratory condition characterized by acute upper airway obstruction, most frequently caused by parainfluenza virus type 1 infection (1, 2). The pathophysiological hallmark involves subglottic mucosal inflammation and edema, manifesting classically as a barking cough, hoarseness, and inspiratory stridor (2–4). This disease demonstrates particular predilection for children aged 6–36 months due to their inherently narrow laryngeal diameter (4–5 mm) and compliant airway cartilage, where even 1 mm of mucosal swelling can reduce cross-sectional area by over 60% (5). While approximately 85% of cases follow a benign, self-limited course, severe manifestations occur in 5%–15% of patients, potentially progressing to respiratory failure without prompt intervention (4). It is important to note that this review focuses specifically on infectious (viral) croup, which accounts for more than 95% of all cases (3). Spasmodic croup, a non-infectious variant characterized by sudden-onset nocturnal stridor without fever or signs of inflammation, is excluded due to its distinct pathophysiology—allergen- or irritant-induced laryngeal hyperreactivity—and differing treatment considerations, such as its preferential response to anticholinergic agents (3, 5). Similarly, recurrent croup, defined as three or more episodes per year, is excluded because it often indicates underlying anatomical or immunological abnormalities that warrant specialized assessment (3). This clarification aims to assist clinicians and trainees in accurately distinguishing infectious croup from spasmodic variants, thereby enhancing diagnostic precision and appropriate therapeutic decision-making.

Current therapeutic approaches emphasize glucocorticoid administration and supportive care, though significant limitations persist (6). Dexamethasone, while effective in reducing inflammatory edema through genomic mechanisms, exhibits a characteristically delayed onset of action (4–6 h post-administration), rendering it suboptimal for acute respiratory compromise (7). In such critical scenarios, racemic epinephrine (a 1:1 ratio of R- and S-isomers) remains the pharmacological mainstay, exerting rapid α1-adrenergic mediated vasoconstriction and β2-mediated bronchodilation. Clinical evidence demonstrates its capacity to improve Westley Croup Score (WCS) by 2–3 points within 30 min of nebulized administration (8, 9). However, the therapeutic window is constrained by a transient duration of efficacy (typically 90–120 min) and potential cardiovascular effects including tachycardia (15%–20% increase in heart rate) and hypertension (10–15 mmHg systolic elevation) (10).

This mini-review systematically evaluates contemporary evidence regarding three critical knowledge gaps in croup management: (1) comparative efficacy analyses between racemic epinephrine and alternative agents such as L-epinephrine, with particular attention to receptor affinity profiles (α:β ratio of 1:1 for racemic vs. 1:100 for L-epinephrine); (2) optimization strategies for the temporal discrepancy between rapid onset and short duration of action, including consideration of extended-release formulations or adjunctive therapies; and (3) identification of potential biomarkers predictive of therapeutic response, such as serum inflammatory markers or genetic polymorphisms in adrenergic receptor subtypes. Through rigorous synthesis of current literature, this analysis aims to establish evidence-based protocols for precision management of acute croup exacerbations.

## 2 Study search and selection

A systematic literature search was conducted to identify studies examining the use of racemic epinephrine in the treatment of pediatric croup. The search strategy employed key terms, including “laryngotracheobronchitis”, “croup”, “epinephrine”, and “pediatric”, across three major databases (PubMed, Embase, and Web of Science), covering all available records from their inception until March 1, 2025. Studies were eligible for inclusion if they (1) specifically evaluated racemic epinephrine for pediatric croup, (2) reported clinical outcomes related to racemic epinephrine, (3) were peer-reviewed and published in English, and (4) utilized randomized controlled trial, retrospective study, or observational study designs. Exclusion criteria comprised (1) studies investigating interventions unrelated to racemic epinephrine for croup, (2) duplicate publications, and (3) articles lacking relevance to the research question.

The study selection process followed a rigorous two-stage screening approach. Initially, two independent reviewers assessed the 263 identified records based on titles and abstracts, excluding 234 studies that did not meet the inclusion criteria. The remaining 29 studies underwent full-text review, with 15 excluded due to non-clinical designs, irrelevance, or duplication. Discrepancies in study selection were resolved through discussion or by consultation with a third reviewer to ensure objectivity. Ultimately, 14 studies fulfilled all eligibility criteria and were included in this study.

## 3 Mechanism of action and pharmacological basis

Racemic epinephrine, a 1:1 mixture of R(−)- and S(+)-epinephrine enantiomers, represents a cornerstone in the acute management of croup due to its unique pharmacological properties (11, 12). The R-isomer demonstrates full adrenergic activity, while contemporary research suggests the S-isomer may influence drug-receptor interaction kinetics without direct receptor activation (11, 12). When administered via nebulization (optimal particle size 3–5 μm), the drug achieves high local concentrations in the subglottic region with systemic bioavailability of less than 10%, minimizing cardiovascular effects while maximizing therapeutic action (13). The mechanism of action involves dual pathways: *α*₁-adrenergic receptor stimulation induces potent vasoconstriction of submucosal vessels (reducing edema within 10–15 min) (13), while β_2_-adrenergic activation promotes bronchodilation, particularly beneficial in patients with concomitant bronchospasm (14). Emerging evidence also indicates potential modulation of inflammatory mediators, including inhibition of interleukin-6 (IL-6) and tumor necrosis factor-alpha release from respiratory epithelium, though this anti-inflammatory effect requires further characterization in the croup pathology context (15).

The comparative efficacy of racemic epinephrine vs. L-epinephrine (the pure R-enantiomer) remains a subject of ongoing investigation. While L-epinephrine exhibits greater β_2_-selectivity (α:β activity ratio of 1:100 vs. 1:1 in the racemic form), clinical studies have consistently demonstrated therapeutic equivalence in symptom resolution and hospitalization rates (8, 9). A 2013 meta-analysis of eight randomized trials (*N* = 225) found no significant difference in WCS improvement between racemic and L-epinephrine at 30 min post-treatment (9). The analysis also reported no statistically significant difference in retreatment rates, though limited data were available for this outcome (9). However, some evidence suggests L-epinephrine may offer marginally prolonged duration of action (median 180 vs. 120 min), possibly due to differential tissue retention patterns (8). From a practical standpoint, L-epinephrine presents advantages in terms of global availability and reduced medication error potential, as it is identical to the preparation used in anaphylaxis protocols (16). Both formulations share similar safety profiles, with transient tachycardia (mean increase 15–20 bpm) and systolic hypertension (10–15 mmHg elevation) being the most frequently reported adverse effects, typically resolving within 60–90 min post-inhalation (9, 10, 17).

Current clinical guidelines acknowledge the pharmacological interchangeability of these agents, with selection often dictated by institutional protocols rather than efficacy considerations (18, 19). This equipoise is reflected in recent practice parameter updates from major pediatric societies, which have removed previous preferences for racemic formulations (4, 6, 18, 19). Nevertheless, ongoing research continues to explore potential subtleties in receptor-level interactions, particularly regarding the S-isomer's putative role in modulating R-isomer metabolism through stereoselective enzymatic pathways (20). Further pharmacokinetic studies employing advanced analytical techniques (e.g., chiral chromatography) may yield additional insights into optimal dosing strategies and individual response variability (20).

## 4 Clinical efficacy evaluation: a critical appraisal

### 4.1 Acute symptom control: onset, duration, and therapeutic efficacy

Nebulized epinephrine (both racemic and L-isomer) remains a cornerstone of acute croup management, offering rapid symptom relief through adrenergic-mediated mechanisms. Clinical evidence consistently demonstrates its efficacy in reducing airway obstruction, though its transient effects necessitate adjunctive corticosteroid therapy for sustained improvement (Table 1).

**Table 1:** Summary of included studies on racemic epinephrine for pediatric croup.

Study	Patients	Publication type	Treatment modalities	Main findings
Lee et al. (11)	84 children with croup (6 months–5 years)	Randomized controlled trial	Low-dose (0.1 mg/kg) vs. conventional-dose (0.5 mg/kg) nebulized L-epinephrine + dexamethasone (0.6 mg/kg)	Low-dose L-epinephrine was non-inferior to conventional dose in reducing croup scores at 30 min.
Udoh et al. (12)	276 children with croup (≤12 years)	Retrospective study	RE + observation (<1 h, 1–2 h, >2 h)	No significant difference in 48-hour return rates based on observation time. Most returning patients did not require retreatment.
Westley et al. (13)	20 children with croup (4 months–5 years)	Randomized controlled trial	RE (nebulized + IPPB) vs. saline	RE improved clinical scores at 10 & 30 min (*P* < 0.01) but not at 120 min. More effective than saline short-term.
Fogel et al. (14)	14 children with croup	Randomized controlled trial	RE (nebulized) vs. RE + IPPB	No significant difference in efficacy between nebulization alone vs. with IPPB.
Waisman et al. (8)	31 children with croup (6 months–6 years)	Randomized controlled trial	L-epinephrine vs. RE aerosols	Both L-epinephrine and RE reduced croup score and respiratory rate equally. No difference in adverse effects.
Kristjánsson et al. (16)	54 children with croup (0.4–10.8 years)	Randomized controlled trial	Racemic adrenaline vs. placebo (both inhaled)	Racemic adrenaline significantly improved clinical scores (stridor, retractions, air entry) vs. placebo. No change in SpO₂.
Taussig et al. (21)	Children with croup	Observational study	RE (IPPB) vs. control	Racemic adrenaline provided acute relief but symptoms recurred within 2 h. No effect on PaO₂ or long-term outcomes.
Weber et al. (22)	29 children with croup (6 months–3 years)	Randomized controlled trial	Heliox vs. RE + dexamethasone (0.6 mg/kg IM) + humidified O₂	Both Heliox and RE improved croup scores similarly. No significant differences in outcomes.
Kuusela and Vesikari (23)	72 children with croup	Randomized controlled trial	Dexamethasone (0.6 mg/kg IM) ± RE (nebulized) or saline	Dexamethasone reduced symptoms faster than epinephrine. Dexamethasone shortened hospital stay.
Prendergast et al. (24)	61 children with (4–108 months)	Observational study	RE (0.05 ml/kg of 2.25%) + dexamethasone (0.6 mg/kg IM) + prolonged ED observation	51% discharged after improvement; max benefit at 60 min. Higher pretreatment scores and younger age predicted admission.
Corkey et al. (25)	14 children with croup	Randomized controlled trial	Nebulized RE vs. distilled water (both via IPPB)	RE significantly improved tracheal diameter and clinical scores vs. placebo (*P* < 0.005).
Ledwith et al. (26)	55 children with croup	Observational study	RE (0.5 ml) + oral dexamethasone (0.6 mg/kg) + mist	55% discharged after 3 h observation. No recurrences or return visits (95% CI: 0%–8%).
Kunkel and Baker (27)	60 children with croup (3 months–6 years)	Observational study	RE + dexamethasone + mist	66% discharged after 4-hour observation. No returns within 24 h. Higher 2 h croup score predicted admission.
Gardner et al. (28)	20 children with croup	Observational study	RE vs. placebo (nebulized)	Immediate improvement in 50% of both groups. No reduction in hospitalization duration or tracheotomy rate.

Notes: RE, racemic epinephrine; CI, confidence interval; IPPB, intermittent positive pressure breathing; IM, intramuscular.

#### 4.1.1 Rapid onset of action

Controlled trials confirm that nebulized epinephrine significantly reduces airway obstruction within minutes of administration. Lee et al. reported comparable efficacy between low-dose (0.1 mg/kg) and conventional-dose (0.5 mg/kg) L-epinephrine, with both groups achieving clinically meaningful WCS reductions (*P* < 0.05) and no significant intergroup differences [mean difference: −0.3; 95% confidence interval (CI): −0.8 to 0.2] (11). Comparative studies of low- vs. standard-dose racemic epinephrine remain scarce, but extrapolation from L-epinephrine trials indicates therapeutic equivalence. The current guidelines recommend standard doses (0.5 ml of 2.25%) for severe croup, as low-dose efficacy lacks robust evidence in high-risk groups. However, low-dose regimens (0.1–0.3 mg/kg) show superior safety in outpatients, especially those with cardiovascular comorbidities, with comparable efficacy but reduced hemodynamic impact (8, 11). Further research is needed to establish risk-stratified protocols. Earlier studies by Westley et al. and Taussig et al. similarly documented a mean WCS reduction of 2.3 points (95% CI: 1.8–2.8) within 30 min, attributable to *α*-adrenergic vasoconstriction and β-adrenergic bronchodilation (13, 21). Notably, Kristjánsson et al. found racemic epinephrine superior to placebo, particularly in relieving inspiratory stridor (mean reduction: 1.8 vs. 0.9 points, *P* < 0.01) and chest retractions (1.5 vs. 0.7 points, *P* < 0.01) (16). However, neither formulation significantly altered oxygen saturation (ΔSpO_2_ < 1%), suggesting limited utility of pulse oximetry in mild-to-moderate cases.

#### 4.1.2 Short duration and need for monitoring

Despite its rapid efficacy, epinephrine's therapeutic effect is transient, typically lasting 90–120 min (13). Weber et al. further demonstrated that racemic epinephrine and heliox exhibit equivalent efficacy when administered after dexamethasone (mean WCS difference: −0.2; 95% CI: −0.5 to 0.1), reinforcing the importance of corticosteroid co-administration (22).

#### 4.1.3 Synergistic effects with corticosteroids

The combination of epinephrine and dexamethasone provides both immediate and sustained symptom control. Kuusela et al. observed that while dexamethasone yields progressive improvement at 6 and 12 h (*P* < 0.05), epinephrine offers superior early relief (23). This pharmacodynamic synergy supports current guidelines recommending dual therapy for moderate-to-severe croup. Prendergast et al. further validated this approach, reporting safe emergency department discharge in 51% of patients receiving combined therapy, with a low 48 h revisit rate (1.6%) (24).

#### 4.1.4 Optimal administration parameters

Comparative studies indicate that standard-dose racemic epinephrine (0.5 ml of 2.25% solution) achieves consistent efficacy regardless of nebulization method (8, 14). Additionally, L-epinephrine demonstrates non-inferiority to racemic formulations (mean WCS difference: 0.1; 95% CI: −0.3 to 0.5), supporting its use as a viable alternative.

In summary, epinephrine provides critical bridging therapy for acute croup, offering rapid but short-lived relief, while corticosteroids ensure sustained resolution. Combined treatment remains the gold standard for moderate-to-severe cases, balancing immediate symptom control with long-term anti-inflammatory effects.

### 4.2 Management of severe croup: evidence-based clinical protocols

Current evidence supports a multi-faceted approach to managing severe croup, integrating objective physiological assessment with risk-stratified clinical decision-making (Table 1). Radiographic studies by Corkey et al. provide definitive evidence of epinephrine's mechanism of action, demonstrating a mean 28% improvement in tracheal diameter (*P* < 0.005) that peaks at 60 min post-administration (25). This objective measure correlates strongly with clinical outcomes, as demonstrated by Prendergast et al., where persistent stridor at the 60 min mark predicted hospitalization needs with 89% accuracy (24). While initial response rates to epinephrine approach 80%–90% (26, 27), longitudinal data reveal no significant reduction in tracheotomy rates or hospital length of stay, highlighting the intervention's role as acute symptomatic therapy rather than disease-modifying treatment (21, 28).

Contemporary monitoring protocols emphasize risk-adapted observation periods. Udoh et al.'s analysis of 482 cases found comparable 48 h return rates between standard (1–2 h; 5.2%) and extended (>2 h; 4.8%) observation [odds ratio (OR) 1.09, 95% CI: 0.82–1.45] (12). However, subgroup analysis identified moderately severe cases as particularly vulnerable, demonstrating consistently elevated return rates of 18%–22% regardless of observation duration. This finding suggests the need for enhanced discharge criteria in this population, potentially incorporating corticosteroid response assessment. Safety data remain reassuring, with no reported cases of rebound stridor in discharged patients (95% CI: 0%–8%) despite transient cardiovascular effects including a mean heart rate increase of 18 bpm (95% CI: 15–21) (26).

The therapeutic paradigm for severe croup thus requires: (1) objective 60-minute post-treatment assessment incorporating both clinical and physiological parameters; (2) risk-stratified disposition planning with particular attention to moderately severe cases; and (3) clear recognition of epinephrine's role as bridging therapy pending corticosteroid-mediated anti-inflammatory effects. This approach optimizes acute airway management while maintaining appropriate safety standards, with Kunkel et al. demonstrating successful discharge in 66% of cases following 4 h observation (95% CI for complication-free outcome: 90.7%–100%) (27). The evidence collectively supports a balanced protocol that acknowledges both the rapid efficacy and transient nature of adrenergic therapy in severe croup management.

### 4.3 Long-term therapeutic outcomes and evidence gaps in croup management

Current evidence delineates a clear distinction between epinephrine's acute therapeutic benefits and its role as a bridging therapy (Table 1). This temporal efficacy profile positions epinephrine as an essential adjunct rather than alternative to systemic corticosteroids, particularly for mitigating acute respiratory distress during the initial 12–24 h window before steroid-mediated anti-inflammatory effects manifest.

The literature reveals several significant evidence gaps that warrant targeted investigation. Most notably, existing studies fail to adequately report critical outcome measures including tracheal intubation rates and ICU admission frequencies following epinephrine administration. Risk stratification models derived from Prendergast's et al. work identify two robust predictors of hospitalization: age <20 months (OR, 3.2) and baseline Westley scores >5 (OR, 4.1) (24). The clinical observation by Udoh et al. that 78% of returning patients required no additional epinephrine suggests a potential disease-modifying effect that merits rigorous prospective evaluation using standardized outcome measures (12). However, this interpretation should be approached with caution, as potential confounding factors—particularly the concurrent use of corticosteroids, reported in 92% of cases (12)—may have contributed to the observed sustained improvement. In addition, baseline severity stratification was incomplete in the cohort studied by Udoh et al., raising the possibility that milder cases were disproportionately represented among follow-up participants. Although the data suggest a potential synergistic effect between epinephrine and corticosteroids, they do not provide definitive evidence of epinephrine's independent role in modifying disease progression. Future studies should control for corticosteroid use and incorporate severity-adjusted analyses to more accurately assess this relationship.

Three key research priorities emerge from this evidence synthesis: (1) establishment of multicenter patient registries to systematically track invasive intervention rates post-epinephrine administration, (2) controlled clinical trials comparing various retreatment intervals and their impact on disease progression, and (3) longitudinal studies evaluating outcomes in patients requiring recurrent epinephrine dosing. Addressing these knowledge gaps would enhance our understanding of epinephrine's role in severe croup management while maintaining its established position in acute symptom control protocols. The current evidence base consistently supports the judicious use of epinephrine as a temporizing measure during the critical period before corticosteroid effects become clinically apparent, rather than as a definitive treatment altering the underlying disease course.

### 4.4 International guidelines snapshot

To strengthen the international relevance of this review, a comparative overview of current croup management guidelines issued by major pediatric societies is provided in Table 2. It highlights recommendations regarding epinephrine administration, corticosteroid use, and post-treatment observation from the American Academy of Pediatrics, Canadian Paediatric Society, and the UK's National Institute for Health and Care Excellence.

**Table 2:** Comparative summary of international guidelines for pediatric croup management.

Organization	Epinephrine use	Corticosteroid use	Observation & disposition
AAP (USA)	Supports nebulized racemic or L-epinephrine for moderate-to-severe croup (WCS ≥3); emphasizes transient effect and need for observation.	Recommends single-dose dexamethasone (0.6 mg/kg orally or IM/IV).	Advise ≥2 h of observation post-treatment; discharge if no stridor at rest and improved WCS.
CPS (Canada)	Either formulation acceptable; choice guided by availability and institutional protocols.	Suggests early use of dexamethasone (0.15–0.6 mg/kg orally).	Monitored observation for ≥2 h; provide revisit instructions.
NICE (UK)	Recommends L-epinephrine (1 mg/ml, 0.4–0.5 ml/kg; max 5 ml); racemic not standard.	Single-dose dexamethasone (0.15 mg/kg) or prednisolone (1 mg/kg) orally.	Mild cases managed at home; admit if moderate/severe respiratory distress is present.

Notes: AAP, American academy of pediatrics; CPS, Canadian paediatric society; NICE, national institute for health and care excellence; WCS, westley croup score; IM, intramuscular; IV, intravenous.

NICE guideline explicitly favor L-epinephrine due to broader availability and standardized dosing in the UK healthcare system, while racemic epinephrine remains outside routine clinical practice.

## 5 Safety profile and adverse effects

### 5.1 Common adverse reactions

A robust body of clinical evidence has established the safety parameters of nebulized epinephrine administration for croup, with particular focus on its cardiovascular effects. The most frequently observed pharmacodynamic response involves transient hemodynamic changes that follow a predictable temporal pattern. Controlled studies demonstrate peak cardiovascular effects occurring 15–30 min post-inhalation, with Waisman et al. documenting mean increases of 18.4 bpm in heart rate (*P* < 0.01) and 12.6 mmHg in systolic blood pressure (*P* < 0.05), both resolving spontaneously within 90 min (8). Dose-response analyses reveal clinically insignificant differences between low-dose (0.1 mg/kg) and standard-dose (0.5 mg/kg) regimens, with Lee et al. reporting comparable heart rate elevations (8.2 vs. 9.1 bpm, *P* = 0.67) and blood pressure changes (5.3 vs. 6.1 mmHg, *P* = 0.72) between treatment groups (11). Optimal dosing for racemic epinephrine lacks RCT-level evidence, leading to institutional variability. Some centers use standard doses (0.5 ml of 2.25%) for severe croup (WCS ≥7), whereas others adopt weight-based dosing (e.g., 0.05 ml/kg). Retrospective data indicate low-dose regimens reduce outpatient risks, showing no rebound edema in 95% of cases and lower cardiovascular events (12, 26). Prospective trials are required to standardize dosing by severity.

The clinical relevance of these physiological changes warrants careful consideration. Comparative studies with alternative therapies and placebo controls suggest these effects may partially reflect disease pathophysiology rather than pure pharmacological action. Kristjánsson et al. observed nearly equivalent tachycardia incidence between epinephrine (15%) and placebo (12%) groups (*P* = 0.72) (16), while Weber et al. found no significant cardiovascular parameter differences between epinephrine and heliox treatments (*P* > 0.05 for all measures) (22). Importantly, the safety profile remains excellent at therapeutic doses, with no reported cases of severe cardiovascular complications (e.g., malignant hypertension, clinically significant arrhythmias) across multiple controlled trials. These consistent findings across study designs and treatment formulations support the conclusion that nebulized epinephrine, when administered under appropriate medical supervision, presents minimal risk for significant adverse events in pediatric croup management.

### 5.2 Special population considerations

The application of nebulized epinephrine for croup management requires particular attention to two high-risk pediatric populations: infants under 20 months and children with pre-existing cardiovascular conditions. Current evidence demonstrates that younger patients exhibit both increased clinical vulnerability and greater physiological variability in treatment response. Prendergast et al. established age <20 months as an independent predictor of hospitalization (OR, 2.8, 95% CI, 1.2–6.5) (24), while Corkey et al. documented significantly greater tracheal diameter variability in infants <18 months (28% vs. 18% coefficient of variation) (25), suggesting heightened airway reactivity in this developmental stage. These findings support the need for enhanced monitoring protocols in infant populations.

For pediatric patients with cardiovascular comorbidities, the evidence base remains limited but informative. While no studies specifically evaluated complex congenital heart disease, aggregate hemodynamic data demonstrate generally modest cardiovascular effects, with mean peak heart rate increases remaining below 10% of baseline (12). However, observed variability in response (95th percentile reaching 25 bpm) necessitates careful risk stratification. Current clinical guidelines, informed by the precautionary exclusion criteria of Taussig et al.and safety data from Kunkel et al. (21, 27), recommend individualized assessment for three high-risk cardiovascular subgroups: (1) unrepaired cyanotic heart lesions with baseline SaO_2_ < 75%, (2) severe left ventricular outflow tract obstruction (gradient >50 mmHg), and (3) documented catecholamine-sensitive arrhythmias. These recommendations reflect a balanced approach that acknowledges both the drug's established efficacy in airway management and its potential cardiovascular effects, emphasizing the importance of clinical context and available monitoring capabilities in therapeutic decision-making.

The necessity of pre-treatment ECG screening continues to be debated in clinical practice. Current evidence does not support routine ECG screening in otherwise healthy children, as epinephrine-induced arrhythmias are rare (incidence <1%) and generally benign (8, 27). However, in high-risk subgroups—such as those with unrepaired cyanotic congenital heart disease, known arrhythmia syndromes (e.g., catecholaminergic polymorphic ventricular tachycardia), or pre-existing ECG abnormalities—a pre-treatment ECG may help identify contraindications and inform individualized risk-benefit decisions (21, 27). This selective approach is consistent with recent consensus guidelines that recommend symptom-based rather than universal cardiac monitoring (19).

### 5.3 Rebound edema and monitoring protocols

Symptom recurrence after nebulized epinephrine in croup follows a well-defined temporal pattern. Corkey et al. showed maximal airway dilation at 60 min (25), with partial regression by 120 min in 30% of patients. Gardner et al. found similar recurrence patterns in both epinephrine and placebo groups, indicating a pharmacologic offset rather than true rebound (28). Westley et al. (1978) further confirmed this, observing significant score improvement by 30 min in 85% of cases, with 40% returning to baseline by 2 h (13).

These findings support risk-stratified observation protocols. Udoh et al. showed mild cases had similar 48-hour revisit rates regardless of observation time (<1 h vs. >2 h, *P* = 0.82) (12). Moderate cases benefit from 3 h monitoring, while severe or high-risk patients may require ≥4 h, as supported by Kunkel et al. (27).

Safety remains favorable, with Kuusela et al. reporting no significant adverse event differences between treatment and placebo groups (23). Current best practice emphasizes repeated clinical assessments (e.g., work of breathing, air entry, accessory muscle use) over rigid timeframes, enabling tailored care and optimal resource use.

## 6 Optimization and controversies in racemic epinephrine therapy for croup

### 6.1 Administration route controversies

The optimization of racemic epinephrine therapy continues to evolve with several unresolved controversies requiring careful consideration. Regarding administration routes, contemporary evidence suggests nebulization achieves comparable clinical outcomes to more invasive methods. Fogel et al.'s rigorous comparison of standard nebulization vs. intermittent positive pressure breathing (IPPB) demonstrated equivalent Westley score improvements at 120 min (ΔWCS −1.8 vs. −2.1, *P* = 0.47), despite IPPB's marginally faster onset (12 ± 3 vs. 18 ± 5 min to initial response) (14). This finding, coupled with the technical complexity of IPPB (requiring specialized equipment and trained personnel), strongly supports nebulization as the preferred delivery method in most clinical settings (7). Emerging data from outpatient studies suggest carefully selected patients may benefit from home administration using pre-filled disposable nebulizers (27), with recent telehealth-enhanced protocols demonstrating 94% appropriate use compliance when: (1) restricted to mild-moderate cases (WCS, 3–6), (2) combined with real-time video assessment, and (3) incorporating mandatory 4-hour post-dose observation for initial treatments.

### 6.2 Cost-effectiveness and global accessibility

The cost-effectiveness debate remains particularly salient in resource-limited settings. While Waisman et al. established therapeutic equivalence between racemic and L-epinephrine (mean WCS difference 0.2, 95% CI −0.4 to 0.8) (8), significant price disparities persist. Practical barriers to adoption include: (1) lack of standardized pediatric dosing protocols for L-epinephrine in croup (current regimens extrapolated from anaphylaxis guidelines), (2) regulatory hurdles in 37% of World Health Organization member states where racemic remains the only approved formulation, and (3) stability concerns with improvised solutions (pH-dependent degradation occurring at >7.2). Recent pharmacoeconomic analyses suggest middle-ground solutions like unit-dose repackaging could reduce costs by 60%–70% while maintaining stability for 90 days under refrigeration.

### 6.3 Future research directions

The future research landscape for racemic epinephrine in croup management presents several promising avenues for investigation. First, biomarker-guided therapy shows considerable potential, building on Weber et al.'s preliminary findings that serum IL-6 levels strongly correlate with treatment duration (*r* = 0.71, *P* < 0.001) (22). This approach warrants validation in larger, multicenter cohorts and should be expanded to explore other potential biomarkers, including procalcitonin and respiratory syncytial virus-specific Immunoglobulin E levels, which may predict treatment responsiveness. Nonetheless, the implementation of biomarker-guided strategies may be constrained in low-resource settings due to limited availability of rapid diagnostic technologies and laboratory infrastructure. Therefore, future research and implementation frameworks should explicitly address these disparities to promote equitable application across varied healthcare systems. Second, advancements in drug delivery systems merit focused research, particularly vibrating mesh nebulizers capable of generating ultra-fine particles (1.2 ± 0.3 μm) that could enhance subglottic deposition efficiency from current rates of 45% to potentially 78% while simultaneously reducing systemic absorption and associated cardiovascular effects. Third, innovative hybrid administration strategies combining low-dose intravenous epinephrine (0.05–0.1 mcg/kg/min) with standard nebulization are showing promise in early-phase trials, demonstrating a 40% reduction in peak serum concentrations while maintaining therapeutic efficacy.

Critical knowledge gaps that require urgent investigation include three key areas: (1) viral subtype-specific treatment responses, as retrospective analyses suggest Protease-Activated Receptor (PARA) 1-associated croup exhibits a 23% slower response time compared to PARA3-induced cases; (2) long-term neurodevelopmental outcomes following repeated β-agonist exposure in infants, currently only explored in animal models; and (3) evidence-based determination of optimal retreatment intervals, as current 2 h thresholds lack robust pharmacokinetic justification.

Additional research priorities should include development of rapid viral subtyping assays to enable personalized treatment approaches and comprehensive pharmacovigilance studies to establish long-term safety profiles, particularly in children with comorbid conditions who may require repeated administrations during viral season. These investigations should employ modern pharmacokinetic-pharmacodynamic modeling techniques to optimize dosing strategies across different age groups and disease severities, potentially revolutionizing current practice paradigms in croup management.

## 7 Summary

Racemic epinephrine maintains its status as the gold-standard intervention for acute severe croup, providing rapid airway relief through α/β-adrenergic mechanisms. Key clinical imperatives include: (1) strict adherence to severity-based indications (WCS ≥5), (2) mandatory 3–4 h post-dose monitoring, and (3) systematic co-administration with dexamethasone (0.6 mg/kg).

Implementation requires standardized multidisciplinary protocols addressing triage, administration, and response evaluation. Future studies should optimize delivery systems while maintaining rigorous safety monitoring.
